# Translational models of adaptive and excessive fighting: an emerging role for neural circuits in pathological aggression

**DOI:** 10.12688/f1000research.18883.1

**Published:** 2019-06-25

**Authors:** Herbert E. Covington, Emily L. Newman, Michael Z. Leonard, Klaus A. Miczek

**Affiliations:** 1 Department of Psychology, Tufts University, Medford, 530 Boston Ave, 02155, MA, USA; 2 Department of Neuroscience, Tufts University, Boston, 136 Harrison Ave, 02111, MA, USA

**Keywords:** Aggression, violence, plasticity, phylogeny, animal models, reward, circuitry

## Abstract

Aggression is a phylogenetically stable behavior, and attacks on conspecifics are observed in most animal species. In this review, we discuss translational models as they relate to pathological forms of offensive aggression and the brain mechanisms that underlie these behaviors. Quantifiable escalations in attack or the development of an atypical sequence of attacks and threats is useful for characterizing abnormal variations in aggression across species. Aggression that serves as a reinforcer can be excessive, and certain schedules of reinforcement that allow aggression rewards also allow for examining brain and behavior during the anticipation of a fight. Ethological attempts to capture and measure offensive aggression point to two prominent hypotheses for the neural basis of violence. First, pathological aggression may be due to an exaggeration of activity in subcortical circuits that mediate adaptive aggressive behaviors as they are triggered by environmental or endogenous cues at vulnerable time points. Indeed, repeated fighting experiences occur with plasticity in brain areas once considered hardwired. Alternatively, a separate “violence network” may converge on aggression circuitry that disinhibits pathological aggression (for example, via disrupted cortical inhibition). Advancing animal models that capture the motivation to commit pathological aggression remains important to fully distinguish the neural architecture of violence as it differs from adaptive competition among conspecifics.

The adaptive significance of aggressive acts has been critically examined for more than a century now ^1^, revealing neural substrates of agonistic behaviors, including excitatory and inhibitory amino acids, monoamines, and neuropeptides, in invertebrates, reptiles, fish, rodents, and non-human primates. Such efforts piece together the progression of phylogenetically conserved behaviors beginning with social pursuit and culminating in forceful attacks. The framework provided by these studies on species-typical aggression has allowed us to characterize deviations from adaptive agonistic encounters with the potential to identify the neurobiology of pathological aggression as it pertains to human and veterinary medicine and the criminal justice system. These discoveries guide ongoing and future scientific endeavors that will clarify the neuroplastic events that ultimately promote maladaptive aggression and violence.

## Adaptive aggression

Defense of one’s territory, resources, social position, and group identity depends on the activation of neural circuits that overlap and interact with those involved in many other prominent adaptive social behaviors, including sex, parenting, bonding, and play ^2– 4^. Mammalian aggression is rooted in limbic and diencephalic circuits, including connections between the medial amygdala (MeA), preoptic area, bed nucleus of the stria terminalis (BNST), lateral septum (LS), ventral portion of the premammillary nucleus (PMv), ventral lateral portion of the ventromedial hypothalamus (VMHvl), supramammillary nuclei (SUM), anterior hypothalamic nucleus, and paraventricular nucleus of the hypothalamus ^5^. Evidence suggests that these areas collectively encompass a hierarchical role in the generation of attack sequences, and they also appear in a generalized social network in the brain ^6^.

For many species, pheromonal cues from hostile social targets are coded by the depolarization of Gα _O_-containing vomerosensory neurons (VSNs) in the basal vomeronasal organ ^7^, triggering a distinctive pattern of activation across cortical, limbic, and brain stem nuclei to initiate or inhibit an attack ^8^. Here, evidence favors parallel processing of pro- and anti-social information to yield an adaptive behavioral output. Co-activation of Gα _i_-positive VSNs by alternative non-volatile pheromonal signals can modulate the pro-aggressive actions of Gα _O_-positive cells ^9, 10^.

Sensory signals carried by VSNs terminate in the accessory olfactory bulb where the topographical organization of odor information is relayed to the MeA and other limbic structures (for example, BNST) according to a social stimulus–dependent activational pattern ^11^. Inputs from the amygdala, BNST, and LS to the hypothalamus further coordinate the production of autonomic and somatic attack elements ^12– 16^. The PMv provides excitatory stimulation of the murine SUM and VMHvl, which modulate attack duration and the total number of attacks during a fight, respectively ^17^. Steroid receptor–expressing neurons (that is, ESR1 ^+^ cells) in the VMHvl appear to shape the strength and quality of these attacks ^18– 20^. Indeed, the excitability of hypothalamic nuclei adapts to repeated fighting experiences ^21^. Specifically, daily bouts of aggression fine-tune ensembles of ESR1 ^+^ VMHvl neurons in mice, suggesting that cell populations active during the execution of a fight are reciprocally modified by these behavioral outputs ^22^. Significant measures of plasticity, including changes in catecholamine uptake, have also been demonstrated in cerebral cortex of mice with only a single aggressive experience ^23, 24^.

Winning social confrontations or territorial fights largely depends on the functional integrity of these limbic and hypothalamic brain areas. The mesocorticolimbic dopamine system further encodes information about the outcome of these social encounters. This action–outcome link is evident in the maintenance of social status and contributes to the motivation to engage in or instigate future fights ^17, 25– 29^. The expression of neuropeptides and their receptors (that is, corticotropin-releasing factor, oxytocin, and vasopressin) that modulate monoaminergic neurons is equally important for stabilizing patterns of aggressive behavior ^30, 31^. These fighting-induced patterns of neural activity are accompanied by generalized increases in arousal and by elevations in serum corticosterone concentrations indicating instant sympathetic activity followed by glucocorticoid activation. Furthermore, these systems are influenced by endogenous anabolic steroids. Testosterone levels in male marine iguanas of the Galápagos rise significantly during the mating season, when their hostility toward other males also escalates; when hormone levels dip, the males cohabitate peacefully. Similar observations for species-typical aggression have been measured in fighting fish, roosters, rodents, and primates ^32– 36^.

Current neuron-specific viral manipulations are guided by evidence from early electrical recording and stimulation work with microelectrodes aimed at cell clusters in the medial and lateral hypothalamus ^37– 42^ as well as the central and periaqueductal gray ^43, 44^. Pioneering work by Chi and Flynn points to a modulatory role of cortical and limbic structures over hypothalamically triggered aggressive behaviors ^45^, including a hippocampal–septal–hypothalamic circuit ^46^. Leroy *et al*. ^47^ recently corroborated and extended this proposal, showing that the dorsal CA2 region of the hippocampus provides excitatory tone over the dorsal LS. This glutamatergic pathway disinhibits the VMHvl through GABAergic modulation of the ventral LS, which was found to directly reduce the tonic activity of VMH ESR1 ^+^ neurons activated during a fight. Efforts like these that dissect the complex circuitry underlying specific motivational, hedonic, and adaptive determinants of aggression will provide critical insight about potentially pathological reactive and cold forms of agonistic behaviors.

Evidence for a hierarchical neural organization of species-typical aggression is further supported by studies targeting midbrain structures, including the central gray at the level of the superior colliculus. Recordings from the dorsal periaqueductal gray (PAG) reveal its functional activation during fights and electrical stimulation of this region rapidly generates intense affective defense ^48, 49^. Tracing studies have identified descending second-order glutamatergic PAG projections to the pontine nucleus, raphe magnus, and locus coeruleus that modulate arousal, sympathetic tone, and motor aspects of the well-characterized feline defensive attack ^44^. Ascending attack-promoting tracts densely target areas of the VMH, which, as mentioned above, also coordinates elements of attack performance through reciprocal PAG innervations. Much less is known about upstream telencephalic modulators of species-typical aggression, although changes in functional activity within the neocortex have been observed after fighting in rodents and hamsters ^50– 52^. In addition, the initiation of attack, execution patterns, and cessation of an aggressive bout can be significantly modulated by descending cortical projections targeting limbic and brain stem areas. Such observations point to a possible source of input for “top-down” dysregulation of what is typically adaptive fighting ^53^.

Aggressive behaviors, even in contests without physical contact, can be advantageous and may be the basis for the “winner effect”, particularly when resources are scarce. When aggressive displays lead to physical attacks, dominant and subordinate profiles emerge quickly and often result in the formation of a social hierarchy ^54^. Such encounters are a normal part of daily intercourse for most gregarious animals. Thus, it is necessary to consider efficient fighting in the context of a specific species’ adaptive behavioral repertoire. Understanding the cortical and subcortical mechanisms that are fundamental to orchestrating species-typical adaptive aggression is essential for characterizing the pathological origins and neurobiology of excessive aggression ^55, 56^. Effective models of pathological aggression should, in simple terms, be capable of identifying the neural processes that motivate organisms to fight excessively under conditions that would not typically produce intense or prolonged attacks. It is important to consider that even the most excessive forms of fighting can be circumstantially adaptive.

## Modeling pathological aggression

According to classic ethological perspectives, pathological aggression does rarely manifest beyond humans, as aggression is considered a necessary means for survival ^57^. But in the 1990’s striking reports of intragroup coordinated attacks between neighboring chimpanzees provided evidence to the contrary ^58^. Tanzanian chimpanzee *raids* on neighboring troops living in bordering territories present themselves as excessive, antisocial, and violent ^58^. As reviewed by de Boer ^59^, rodent and other animal models of excessive aggression are characterized by operational criteria that include elements that are impulsive (short latency to attack), excessive (increased levels of attack), and socially atypical (injurious attack topography, disregard for submissive signals, and indiscriminate social targeting). In line with these deviations in behavior, unique patterns of functional neural activation are apparent in comparisons between potentially pathological and species-typical aggressors. For example, artificial selection of male mice with short attack latencies generates a population of excessively aggressive animals that exhibit atypical patterns of activation (that is, c-Fos expression) in the medial prefrontal cortex, central amygdala, and the aforementioned ventral lateral portion of the PAG and VMHvl after a fight ^60^.

Clinical evidence strongly supports a top-down theory for the occurrence of violence ^61^. Transcranial direct current stimulations of the human dorso-lateral prefrontal cortex decrease intentions for physical or sexual assault in healthy women and men ^62^. Evidence from imaging studies further demonstrates that deficits in cortical inhibition promote hyper-reactive amygdaloid responses to aggressive words, which in turn may facilitate maladaptive aggression ^63^. In such cases, the impulse to act aggressively may be less of a choice and more the product of hardwired cortical dysfunction. Experimental models incorporating mice corroborate the occurrence of cortically mediated pathological aggression, whereby brief optogenetic stimulations (20 Hz) of channelrhodopsin or halorhodopsin residing on neurons in the prefrontal cortex bi-directionally control patterns of fighting behavior. In fact, stimulation delays the onset of an attack whereas inhibition of cortical cells facilitates vicious attacks ^64^. A wealth of human studies suggest that areas of the cortex (for example, the anterior cingulate cortex) allow us to resolve emotional and cognitive conflicts via inhibitory control over downstream limbic targets ^61^. However, it should be noted that descending excitatory pathways targeting hypothalamic attack areas can also facilitate impulsive-like aggressive behaviors at least in rodents. Stimulation of pyramidal neurons that specifically innervate the mediobasal hypothalamus increases the number of offensive attacks, and activation of inputs to the lateral hypothalamus favors attacks directed at vulnerable areas of an opponent’s body ^53^. Although most preclinical studies such as these employ loss- or gain-of-function parameters to determine the relationship between cortical nuclei and downstream limbic targets, it remains important to learn how genetic ^65^, social ^66^, environmental ^67, 68^, and pharmacological ^69^ variables might shape specific nuclei within cortical and subcortical cell groups and perhaps contribute to expressions of pathological aggression. In sum, rigorous experimental analyses of aggressive behaviors following manipulations of cortical regions are of high importance given the clinical evidence.

## Aggression as a reward

Neural circuits that support fighting evolved independently at several phylogenic levels, including insects, fish, reptiles, and mammals ^56, 70^. Therefore, it was unexpected that members of each of these animal species would eagerly work for the opportunity to attack an opponent, indicating that aggression is further controlled by a conserved reward system ^71– 74^. Winning a confrontation is indeed a positive experience that strengthens future fighting, thus serving as a potent positive reinforcer or reward ^75– 77^. The concept of a reward includes at least three separable behavioral components: conditioning, incentive motivation, and a pattern of affective responses (for example, facial expressions and vocalizations) ^78^. As such, when aggression serves as a reward after instrumental conditioning in mice, it appears to differ from normal territorial fighting because it is not dependent on, or necessarily initiated by, olfactory stimuli, nor does it function to suppress other behaviors, like sex. In this case, seeking an opportunity to fight is triggered by conditioned environmental cues, and operant responding is driven by the opportunity to attack a conspecific (that is, an anticipated aggressive outcome). In line with this hypothesis, a direct infusion of the dopamine D1- and D2-like receptor antagonist SCH23390 into the nucleus accumbens selectively disrupts responding for an expected aggression reward ^27^.

**Figure 1.**
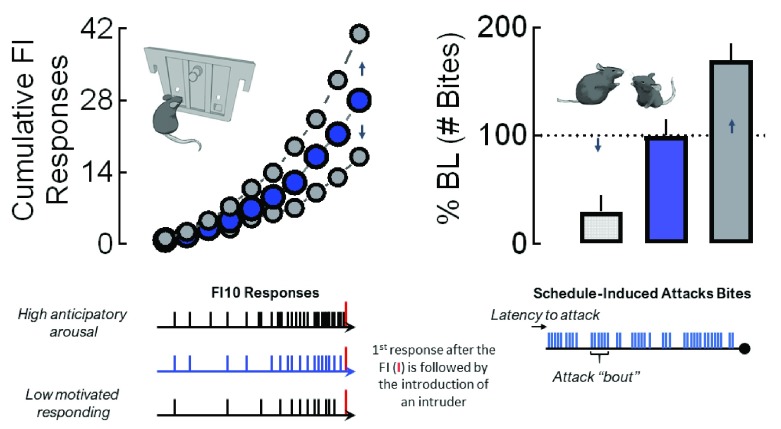
The sequence of experimental events for establishing individual levels of motivated responding for aggression and fighting performance during a daily fixed interval (FI) trial for aggression reward. Prior to their confirmation of territorial aggression, resident male mice are initially housed with females for at least 1 month. In subsequent daily resident–intruder confrontations, each resident male encounters a novel male intruder for less than 5 minutes in the resident’s home cage. After establishing an aggressive phenotype, each resident is trained during a daily FI schedule that is reinforced by the presentation of an intruder (delivered into the resident’s home cage). The duration of the FI is progressively increased from 1 second to 10 minutes over the course of about a month. Resident mice then are allowed to establish a consistent pattern of FI responding. The upper left panel displays typical FI10 scalloped patterns of responding by mice for an aggression reward. Individual responses (tick marks) that are typically generated during a 10-min FI are indicated at the bottom left for varying levels of anticipatory arousal. The first response initiated after the completion of the FI allows the introduction of an intruder into the resident’s home cage. The upper right panel displays one element of fighting behavior (attack bites) that can be examined and potentially altered by experimental manipulations. Other aggressive elements that can be quantitatively and qualitatively examined include the latency to a first attack, number of attack bouts (bottom right), and biting topography (not shown). Changes in appetitive motivational responding can occur with or without alterations in fighting performance.

**Figure 2.**
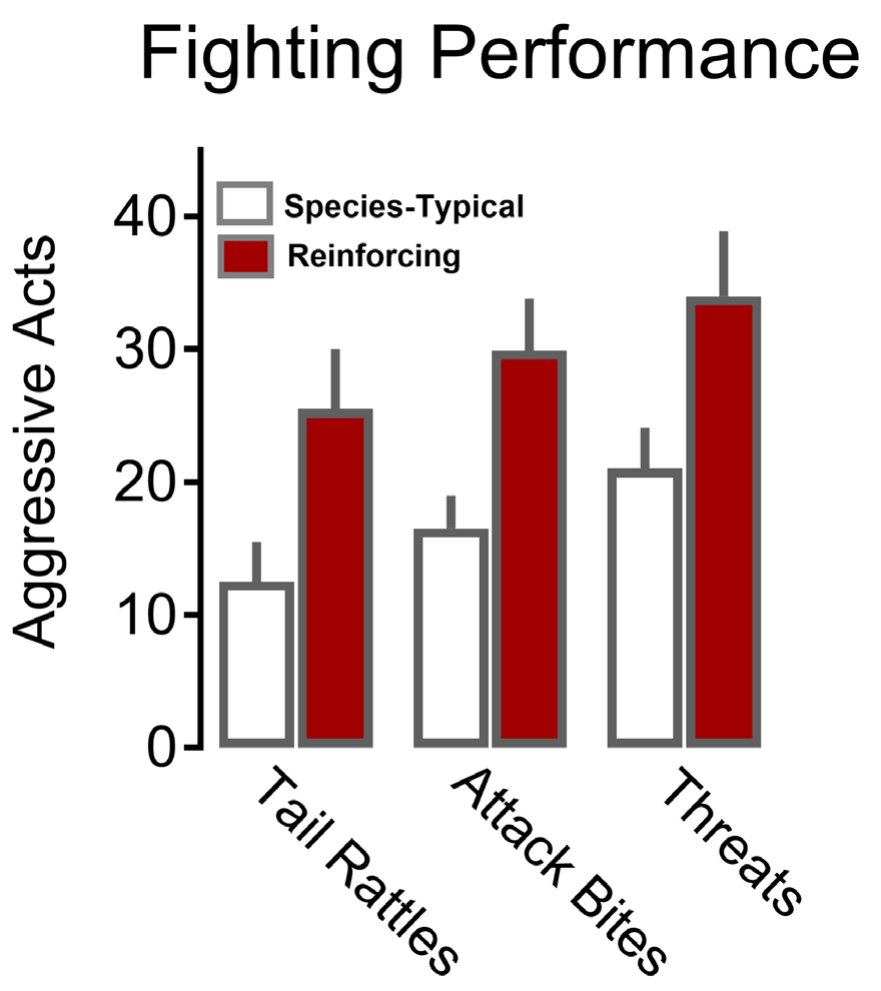
Aggression that serves as a reward is more intense than species-typical fighting. Aggressive behaviors, including tail rattle, sideways threat, and attack bites, are substantially increased when outbred male mice work during a fixed interval schedule for the opportunity to fight a conspecific (dark bars) as compared with species-typical levels of agonistic behavior that occurs during spontaneous aggression (open bars). Adapted from Fish *et al*. ^81^ with permission from Springer-Verlag.

A number of different schedules of reinforcement have been employed to investigate the motivational components of an aggression reward. Patterns and rates of responding during fixed ratio and progressive ratio schedules are perhaps most commonly used for providing an index of the motivational value for a given reinforcer ^79, 80^. However, after completing the demands of a fixed interval (FI) schedule to gain access to a fight, mice display significantly escalated, intense forms of attack that are far more vicious than species-typical fights ^81^ ( Figure 1). These displays of escalated aggression are exemplified by rapid attack latencies followed by a continuous burst of attacks ^82, 83^. Therefore, the pattern of this conditioned form of aggression appears context-specific. Mice conditioned to respond under an FI schedule of aggressive reinforcement display quite normal, species-typical patterns of fighting when an intruder suddenly invades their home cage ( Figure 2).

A significant advantage of FI-conditioned aggression is the sensitive ability to capture the intensity of arousal during the anticipation of a fight. When operant behavior is reinforced by the opportunity to fight, the accelerating rate of operant responding during the interval preceding an aggressive confrontation serves as an index for the motivational state of an animal ^84– 86^. FI schedules allow for very precise dissections of both appetitive motivational and consummatory performance components of an attack ( Figure 1). In fact, clear dissociations between the level of motivation to engage in aggression and the intensity of a fight can be discerned when aggression rewards are controlled by FI schedules, as described below in more detail ^83^.

We speculate that the occurrence of an interaction between arousal and reward expectancy prompts an escalation of fighting once behavioral requirements of the FI schedule are completed. Validated models of frustration similarly escalate the frequency of attacks on an opponent ^87– 89^. Indeed, the frustrative non-reward effect proposed by Amsel and Roussel ^90^ has provided evidence for frustration-induced surges in generalized vigor. FI schedules simply require animals to wait before being reinforced. Close examination of mice performing under an FI schedule reveals a uniquely arousing component that emerges throughout the pre-fight interval. The anticipatory phase elevates corticosterone blood levels ^81^, and there are progressive gains in digging and jumping behaviors from the first to the last minute of the FI ^83^. Likewise, explosive human aggression is preceded by physiological indicators of stress, such as activation of the hypothalamic–pituitary–adrenal axis ^91^ and the emotion of anger ^92^. Thus, despite the anticipation for a reward, some degree of arousal, possibly corresponding to human frustration, is produced by interval schedules, further increasing their translational value when capturing components of explosive clinical aggression. Parametric studies on the physiological and neurobiological components of anticipatory responding for aggression rewards are further warranted.

## Alcohol escalates the urge to fight

**Figure 3.**
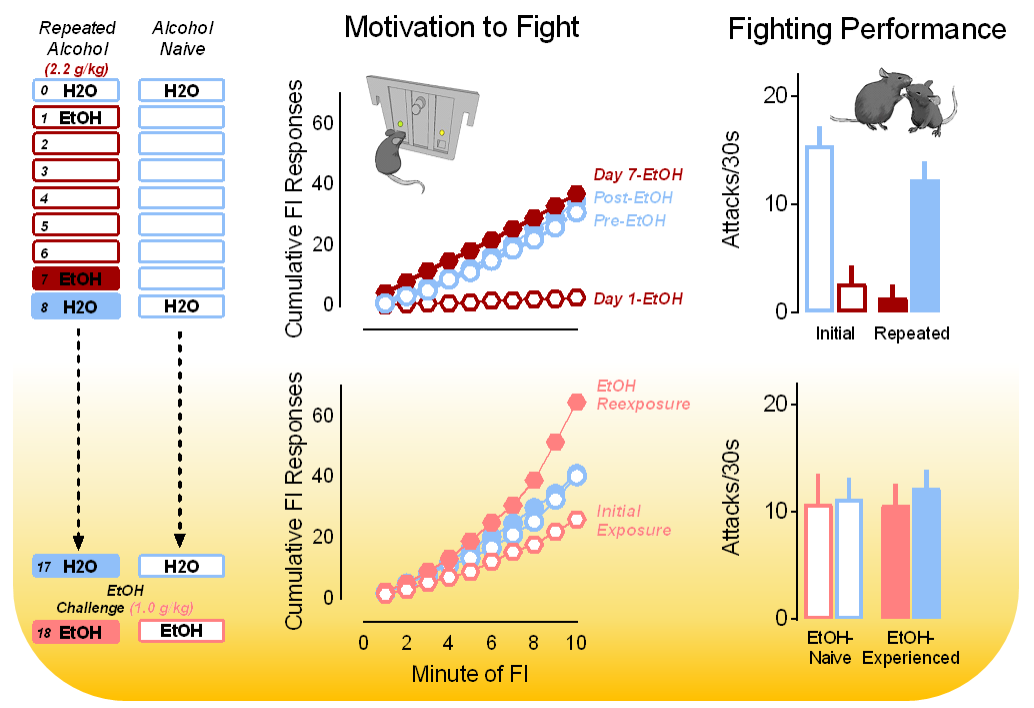
Aggressive motivation escalates with repeated administrations of alcohol and remains persistently sensitized to lower concentrations of alcohol. Fixed interval (FI) response rates (upper middle) are depicted over repeated oral administrations (via gavage) of water or alcohol (2.2 g/kg; top) and 10 days later in response to a dose of water or a low challenge dose of alcohol (1.0 g/kg; bottom middle). Here, the motivation to fight is initially disrupted by alcohol before levels of FI responding recover to baseline. After just seven daily alcohol administrations, the motivation to fight in response to a low dose of alcohol becomes sensitized (bottom middle). Fighting behavior is persistently disrupted over the course of seven consecutive daily administrations of alcohol (2.2 g/kg; upper right) but not significantly affected 10 days later by a challenge dose of alcohol (1.0 g/kg; bottom right). Adapted from Covington *et al*. ^83^ with permission from Frontiers Media SA.

More than half of all violent criminal acts are linked to alcohol consumption, and alcohol-related violence causes significant pain and suffering worldwide ^93– 95^. Such clinical data reveal that cycles of intoxication intensify reactive “hot” acts of violence. Alcohol induces a motivational state that culminates in repeated attempts to pursue violence despite behavioral impairments and uncoordinated motor control ^96, 97^. Most emergency room visits across the globe resulting from alcohol intoxication are due to violence, far outnumbering traffic accidents and incidental mishaps ^98^. In experimental models, alcohol has a wide range of acute and chronic effects on both motivational and consummatory behavioral processes. For example, low doses of alcohol acutely increase fighting performance in mice, rats, and monkeys by increasing the number of attacks and threats without affecting the motivation to fight. In contrast, higher doses of alcohol (>1.8 g/kg) disrupt both motivational and performance aspects of a fight ^83^. Poor fighting performance persists with repeated exposures to alcohol despite a rapid and full recovery of operant responding for the opportunity to fight. This recovery emerges within just a few days, suggesting the development of tolerance to the suppressive effects of alcohol on aggressive motivation. In addition, appetitive response rates actually sensitize with repeated alcohol exposures despite continued disruption of fighting performance ( Figure 3) ^83^. Such data are important given that physical performance is not a necessary component of most violent criminal acts, in which use of a firearm largely exceeds all other acts of aggression ^97, 99^.

The behavioral distinctions between motivational and performance measures suggest that mechanisms promoting aggressive arousal are distinct from those that coordinate the attack. Both conditioned aggression and alcohol-escalated aggression depend on glucocorticoid receptor activation and thus are likely to reflect hot, reactive aggression ^81, 100– 102^. In contrast, cold aggression is associated with a lack of sympathetic arousal and is more frequently linked to predation in animals and psychopathy in humans ^46, 103– 105^. Sites of plasticity in response to repeated administrations of alcohol use (for example, prefrontal cortex, amygdala, extended amygdala, septal nuclei, and monoaminergic terminal regions) may provide neural targets for examining their role in motivating offensive aggression.

## Female aggression

Hillali Matama’s 1974 eye-witness documentation of a Tanzanian chimpanzee party raid was among the first to portray violence in non-human primates which contrasted with popularized accounts of peaceful coexistence and conflict resolution ^58, 106^. In Matama’s account (page 16), the gruesome execution of a lone foraging male chimp was described in detail: “Once again, the victim was an isolated Kahama male—Dé was his name—and the attackers were a gang of *four* (chimpanzees) from Kasekela: three adult males and *one adult female*” ^58^. Each member in this gang of four contributed to the physical assault and brutal killing of Dé. Despite this record and other clear indications of the potential for violence in females across the animal kingdom ^107– 111^, most experimental studies on aggression focus on territorial males. Interestingly, a significant subset of outbred female laboratory mice and rats housed with sterile males will consistently exhibit “rival aggression” toward unfamiliar female intruders ^112, 113^. At least three distinct aggressive phenotypes emerge from a population of regularly cycling outbred Swiss–Webster (that is, CFW and SW) female mice housed with castrated males: rival and gestational aggressors (>65%), exclusively gestational aggressors (>20%), and non-aggressors (~10%) ^113^. These studies add to a rich literature on female gestational aggression ^114, 115^ and defensive maternal aggression ^5, 116, 117^, which are time-locked to hormonal changes associated with pregnancy, parturition, and lactation and serve a specific adaptive function to improve offspring survival.

Male-directed postpartum female aggression increases offspring survival by preventing lactation-concurrent pregnancies and infanticide. In individually housed lactating CFW females, male- and juvenile-directed attacks clearly require activation of estrogen receptor alpha–expressing neurons in the posterior aspect of the VMHvl ^118^. Although females rarely attacked adult female conspecifics under these conditions, activation of VMHvlEsr1 ^+^ cells can promote female-directed aggression. In rodents, hypothalamic inputs from the posterodorsal MeA may modulate the initiation of aggression by relaying threat-related olfactory and pheromonal signals ^119^. It is possible that, for individually housed lactating females, an adult female conspecific does not register as a threat warranting attack. In contrast, the majority of nulliparous CFW females housed with sterile males will readily engage in intense aggression toward adult female conspecifics ^113^. Although gestational and postpartum maternal aggression have clear adaptive purposes, further studies are necessary to identify the neural underpinnings and evolutionary forces that maintain rival aggressors and non-aggressors in a population of nulliparous outbred females.

## Future directions

- Although male animals raised in isolation, including fish, lizards, birds, and most mammals, will readily fight in a species-specific manner (a core component of the innateness of aggression), cellular and molecular data suggest that aggression stems from a neurogenetic network that is extraordinarily plastic. Observations of neural plasticity with increases in aggressive behavioral experiences should be highlighted ^22^. Investigations focused on these processes are providing much-needed insight toward an understanding of the evolution of excessive aggression.
- Quantitative measures that capture the intensity of an aggressive episode such as the latency to attack onset, the attack duration, and the attack frequency are instructive in the early characterization of agonistic behaviors. However, it can be argued that even the most formidable fights are adaptive if they guarantee access to vital reproductive or environmental resources ^120^. Therefore, identifying pathological aggression requires more detailed analyses of attack dynamics and the specific sequence of behaviors exhibited by both the perpetrator and victim. For example, resident male rats will exhibit high levels of aggression toward an intruder conspecific; however, when the submissive intruder displays his ventrum in a supine posture, further attacks are inhibited. Pathological aggression would be evident in an aggressor that continued to attack despite demonstration of the supine posture by the intruder. Lag sequential behavioral analyses may reveal telltale behavioral signals from the subordinate intruder that, under species-typical conditions, should elicit a specific behavioral response from the dominant individual. Similarly, how an aggressive animal attacks an intruder provides valuable information about species-typical and pathological behavior. In mice and rats, most attacks between males are directed toward the posterior dorsum. Atypical placement of attack bites on the vulnerable ventrum of an opponent can reflect pathological aggression and may provide more insight than attack frequency or duration alone ^59, 60, 69, 121, 122^.
- Perhaps more revealing of potentially pathological or violent routines are the patterns of responding that are emitted in anticipation of aggression as a reward. In such cases, the amount of effort exerted before a fight depicts appetitive and anticipatory components of the motivation to fight when fighting does not serve any obvious adaptive function. Mice trained to respond on a task are reinforced by the opportunity to attack an opponent, oftentimes in their own home environment ^82, 83^. Fixed ratio and progressive ratio schedules of reinforcement have been used to assess this motivation in addition to protocols that assess reinstatement of extinguished responding and measures of resistance to punished responding ^71, 123, 124^. Herein, we highlight the use of FI schedules that lead to aggression rewards for several reasons. First, scalloped patterns of FI response curves for individual mice are remarkably stable. We therefore suggest that shifts in FI response rates may indicate sensitive changes in motivational state. Second, mice that are conditioned under these contingencies display excessive aggression only in the context of schedule-controlled aggression. Fighting outside of this context resembles species-typical patterns of fighting ( Figure 2) ^81^. Thus, there is a notable degree of state dependency for this display of escalated aggression in mice that otherwise behave in a species-typical manner. In addition, mice will work extremely hard for the opportunity to fight even when fighting performance has been severely disrupted as a result of alcohol. Not only is this finding translationally significant, but it highlights a unique dissociation between the motivational and consummatory components of a powerful behavioral reward, similar to other natural rewards ^125^.
- One of the urgent tasks is to continue identifying the neurobiological characteristics of cell clusters and pathways mediating aggressive behavior that are conserved across species and translate readily to the human condition in both males and females. It will be important to identify neural mechanisms that control the intensity of behavioral arousal in anticipation of an aggressive opportunity as captured by accelerating rates of responding reinforced by a fight. Hierarchically organized circuits regulate social dominance (for example, amygdala–hippocampal–septal–hypothalamic) as well as defense and subordination ^22, 46, 47, 102, 118, 126– 129^. Yet it remains to be determined how neural circuits that are active during a fight are linked to networks that are active during pre-fight anticipatory arousal. Does aggression induce neural plasticity that promotes a shift in the social brain network toward pathology? It is easy to speculate that the same diencephalic and telencephalic structures that are important for organizing and integrating sensory, motor, endocrine, emotional, and cognitive functions during species-typical aggression also contribute to the production of pathologies in social behavior, including aggression. Alternatively, does the role of an “upstream” mediator determine the level of aggressive output? Cortical structures are vulnerable in the sense that they are (1) influenced by contextual stimuli, (2) highly plastic, and (3) targeted by diverse pharmacological, endocrine, and circadian variables. Experiments that measure and interrogate candidate neural mechanisms during the anticipation of an intense fight will help to clarify the ontogeny of maladaptive aggression. Recent advances in molecular and recording techniques promise to reveal new molecules, cells, circuits, and patterns of temporospatial connectivity between brain regions that contribute to specific types of aggression.
